# Mechanism of impaired glucose tolerance in patients with neoplasia.

**DOI:** 10.1038/bjc.1978.200

**Published:** 1978-08

**Authors:** B. Jasani, L. J. Donaldson, J. G. Ratcliffe, G. S. Sokhi

## Abstract

The disappearance rate (k) of i.v. glucose was measured in cachectic and non-cachectic cancer patients and tumour-free controls. The respective k values were found to be 1.06 +/- 0.27 (mean +/- s.d.), 1.64 +/- 0.34 and 1.63 +/- 0.23. Of the other parameters measured, only plasma albumin level was found to vary significantly amongst the 3 categories, the mean level being the lowest in cachectic cancer patients. The means of total plasma protein, fasting blood glucose and plasma liver enzyme concentrations were similar in the 3 groups. Glucagon, a potent insulin secretogogue, failed to augment the fasting insulin level in cachectic but did so in non-cachectic cancer patients. Taken together, the findings suggest that the reduced glucose tolerance in patients with neoplasia is due to impairment of insulin release exhibited predominantly by ill-nourished advanced cancer patients having a moderate to sever degree of hypoalbuminemia.


*Br. J. Cancer* (1978) **38**, 287

### MECHANISM OF IMPAIRED GLUCOSE TOLERANCE IN PATIENTS WITH NEOPLASIA

B. JASANI, L. J. DONALDSON†, J. G. RATCLIFFE* AND G. S. SOKHI

*From the †Department of Surgery, East Birmingham Hospital, Birmingham B9 5ST, and the *Department of Pathology, Royal Infirmary, Glasgow*

Received 15 March 1978 Accepted 22 May 1978

**Summary.—The disappearance rate (k) of i.v. glucose was measured in cachectic and non-cachectic cancer patients and tumour-free controls. The respective k values were found to be 1·06±0·27 (mean±s.d.), 1·64±0·34 and 1·63±0·23. Of the other parameters measured, only plasma albumin level was found to vary significantly amongst the 3 categories, the mean level being the lowest in cachectic cancer patients. The means of total plasma protein, fasting blood glucose and plasma liver enzyme concentrations were similar in the 3 groups. Glucagon, a potent insulin secretogogue, failed to augment the fasting insulin level in cachectic but did so in non-cachectic cancer patients. Taken together, the findings suggest that the reduced glucose tolerance in patients with neoplasia is due to impairment of insulin release exhibited predominantly by ill-nourished advanced cancer patients having a moderate to severe degree of hypoalbuminemia.**

GLUCOSE intolerance has long been recognized as a manifestation of cancer (Edwards, 1919). It has been shown that the impaired glucose tolerance in neoplasia is independent of age, sex and ethnic origin, and it occurs irrespective of the type of tumour (Glicksman and Rawson, 1956). Tumour-bearing patients, in general, have fasting blood glucose and immunoreactive insulin concentrations similar to those observed in tumour-free controls (Holroyde *et al.*, 1975) even though they have a markedly subnormal disappearance rate of intravenously given glucose (Marks and Bishop, 1956; 1957). The origin of cancer-associated glucose intolerance remains, however, obscure (Glicksman and Rawson, 1956).

Recent studies on malnourished children (Milner, 1971), adults (Smith *et al.*, 1975) and animals (Heard, 1966; Weinkove *et al.*, 1976) conclusively demonstrate that malnutrition can lead to glucose intolerance. In particular the work on malnourished animals (Weinkove *et al.*, 1976) has implicated protein deficiency as an important precursor of impaired carbohydrate metabolism. For instance, weanling rats fed a protein-deficient diet and challenged with i.v. glucose, showed a markedly reduced rate of disappearance of glucose from the blood, compared with normally fed animals. The mean fasting blood-glucose level of the protein-starved rats was found to be similar to that for the controls. This situation is very like that seen in patients with cancer, and makes one think that glucose intolerance in such patients could be due to a form of protein deficiency. To examine this possibility we have studied both well-nourished and ill-nourished cancer patients with respect to their glucose tolerance and protein status.

### METHODS AND MATERIALS

*Patients.*—Of 40 patients studied, 30 had metastatic cancer; 15 being classified as non-

Correspondence to G. S. Sokhi, Department of Surgery, East Birmingham Hospital, Birmingham, B9 5ST.
† Present address: Department of Community Health, University of Leicester, Leicester, LE1 7RH.

cachectic and the rest as cachectic. A patient was defined as cachectic if he displayed anorexia, progressive weight loss and wasting. The type and extent of the malignant disease was diagnosed in all but 2 cases by laparotomy. In these 2 cases, one with carcinoma of the tongue had clinically detectable regional lymph nodes, and one had carcinoma of the bronchus with cerebellar metastases discovered at subsequent autopsy. Any patient with diabetes mellitus or other pancreatic pathology, recent major haemorrhage, sepsis or intestinal obstruction was excluded from the study. The remaining 10 patients were well-nourished routine hospital cases who were included as controls. Every patient had agreed to cooperate in the study, the protocol of which had been initially approved by the local ethical committee.

*Intravenous glucose tolerance test.*—Thirty patients (10 cachectic and 10 non-cachectic cancer patients, and 10 control patients) were starved overnight in preparation for i.v. glucose tolerance test which was performed as follows. A fasting venous blood sample was taken for the estimation of glucose and liver-function. Fifty ml of 50% dextrose solution was then injected over a 2 min period into a relatively large vein. Further blood samples for glucose estimation were taken at 10, 20 and 30 min intervals. Linear regression analysis was performed on the logarithmic values obtained for the blood-glucose samples. From the slope of the regression line obtained, the k value was calculated in each case and expressed as percentage glucose disappearance per minute.

*Glucagon-mediated insulin release.*—Glucagon, a hormone which is known to induce release of pre-formed insulin (Grodsky *et al.*, 1967) was used to study the secretory capacity for pancreatic insulin of 5 cachectic and 5 non-cachectic cancer patients not included in the glucose-tolerance trial. From each patient a fasting venous-blood sample was initially taken for estimation of insulin using a specific radio-immunoassay (Albano *et al.*, 1972). The patient was then subjected to an i.v. injection of glucagon (Eli Lilley & Co.; 0·05 mg/kg body weight) and further blood samples were taken for insulin analysis at 15, 30 and 45 min. The cumulative insulin response was calculated by adding together the differences between the fasting insulin level and the level achieved at each sampling time.

### RESULTS

The detailed characteristics and results of non-cachectic and cachectic cancer

TABLE I.—*Non-cachectic cancer patients*

| Case | Sex & age (years) | Primary site | Extent of metastases | Fasting glucose (mM) | k (%/min) | Albumin (g/l) | Total protein (g/l) | Liver enzymes |
|---|---|---|---|---|---|---|---|---|
| 1 | M 48 | Rectum | Liver | 3·8 | 1·40 | 35 | 66 | Raised |
| 2 | M 55 | Rectum | RLN* | 4·6 | 1·38 | 36 | 63 | Normal |
| 3 | M 72 | Tongue | RLN | 4·1 | 1·43 | 38 | 72 | Normal |
| 4 | M 58 | Lymphosarcoma | RLN | 4·5 | 2·21 | 41 | 68 | Normal |
| 5 | M 74 | Bladder | Spine | 5·3 | 1·68 | 43 | 74 | Normal |
| 6 | M 69 | Colon | RLN | 4·4 | 1·82 | 43 | 70 | Normal |
| 7 | M 54 | Stomach | RLN | 5·0 | 1·73 | 43 | 62 | Raised |
| 8 | M 56 | Colon | RLN | 5·0 | 1·29 | 41 | 67 | Normal |
| 9 | M 61 | Stomach | Liver | 3·7 | 2·12 | 27 | 52 | Normal |
| 10 | F 70 | Ovary | Peritoneum | 5·3 | 1·29 | 31 | 64 | Normal |

* Regional lymph nodes.

TABLE II.—*Cachectic cancer patients*

| Case | Sex & Age (years) | Primary site | Extent of metastases | Fasting glucose (mM) | k (%/min) | Albumin (g/l) | Total protein (g/l) | Liver enzymes |
|---|---|---|---|---|---|---|---|---|
| 1 | M 44 | Colon | Liver; spine | 4·9 | 1·06 | 29 | 73 | Normal |
| 2 | M 75 | Colon | Liver | 4·3 | 0·99 | 31 | 62 | Raised |
| 3 | M 73 | Lung | Brain | 4·2 | 0·71 | 35 | 76 | Normal |
| 4 | F 56 | Stomach | Peritoneum | 5·3 | 1·15 | 30 | 60 | Normal |
| 5 | F 84 | Stomach | RLN | 5·0 | 0·87 | 34 | 59 | Normal |
| 6 | F 72 | Ovary | Peritoneum | 6·1 | 1·17 | 30 | 62 | Normal |
| 7 | M 67 | Oesophagus | RLN | 4·4 | 1·15 | 36 | 61 | Normal |
| 8 | M 62 | Rectum | RLN | 5·5 | 0·92 | 39 | 70 | Normal |
| 9 | M 82 | Rectum | Liver | 6·2 | 1·68 | 30 | 58 | Normal |
| 10 | F 63 | Ovary | Peritoneum | 3·7 | 0·86 | 39 | 62 | Normal |

TABLE III.—*Tumour-free control patients*

| Case | Sex & Age (years) | Diagnosis | Fasting glucose (mM) | k (%/min) | Albumin (g/l) | Total protein (g/l) | Liver enzymes |
|---|---|---|---|---|---|---|---|
| 1 | M 64 | Hydrocele | 4·8 | 1·77 | 35 | 66 | Normal |
| 2 | F 25 | Elective appendicectomy | 4·3 | 1·29 | 40 | 63 | Normal |
| 3 | M 71 | Benign prostatic hypertrophy | 4·4 | 1·24 | 39 | 64 | Normal |
| 4 | F 33 | Duodenal ulcer | 4·7 | 1·75 | 41 | 65 | Normal |
| 5 | M 67 | Benign prostatic hypertrophy | 5·1 | 1·70 | 43 | 74 | Normal |
| 6 | M 62 | Peripheral vascular disease | 3·8 | 1·79 | 45 | 76 | Normal |
| 7 | F 48 | Gall stones | 4·2 | 1·56 | 43 | 69 | Normal |
| 8 | M 60 | Haemorrhoids | 4·2 | 1·56 | 43 | 69 | Normal |
| 9 | M 57 | Inguinal hernia | 4·4 | 1·59 | 41 | 66 | Normal |
| 10 | F 67 | Gastric ulcer | 3·9 | 1·63 | 45 | 69 | Normal |

patients are shown in Tables I and II respectively. The corresponding features of control patients are in Table III. Table IV summarizes the means and standard deviations of the various parameters in the 3 patient categories. It can be seen that the mean k value of cachectic patients differs significantly ($t=4{\cdot}24$; $P<0{\cdot}001$) from that for non-cachectic cancer patients and the controls, which were virtually identical. The mean serum albumin levels for the last 2 categories of patients are seen to be appreciably higher than those for the cachectic cancer patients. For the com-

TABLE IV.—*Summary of data for cachectic and non-cachectic cancer patients and tumour-free controls*

| Patient (No.) | k (%/min) Mean ± s.d. | Albumin (g/l) Mean ± s.d. | Total protein (g/l) Mean ± s.d. | Fasting glucose (mM) Mean ± s.d. | Number of cases with raised liver enzyme levels |
|---|---|---|---|---|---|
| Cachectic (10) | 1·06 ± 0·27 | 33 ± 4 | 64 ± 6 | 5·0 ± 0·8 | 1 |
| Non-cachectic (10) | 1·64 ± 0·34 | 38 ± 6 | 66 ± 6 | 4·6 ± 0·6 | 2 |
| Control (10) | 1·63 ± 0·23 | 42 ± 3 | 68 ± 4 | 4·5 ± 0·3 | 0 |

TABLE V.—*Insulin response to glucagon stimulation*

| Patient | Category | Plasma Insulin (mU/l) 0 min | 15 min | 30 min | 45 min | Cumulative insulin response over 45 min |
|---|---|---|---|---|---|---|
| 1 | Cachectic | 10 | 10 | 10 | 10 | 0 |
| 2 | | 3 | 3 | 15 | 3 | 12 |
| 3 | | 4 | 8 | 8 | 4 | 8 |
| 4 | | 8 | 13 | 14 | 11 | 14 |
| 5 | | 4 | 17 | 8 | 11 | 24 |
| 6 | Non-cachectic | 5 | 35 | 25 | 24 | 69 |
| 7 | | 8 | 36 | 19 | 18 | 49 |
| 8 | | 16 | 34 | 41 | 40 | 67 |
| 9 | | 10 | 38 | 33 | 33 | 74 |
| 10 | | 6 | 64 | 50 | 31 | 127 |

parison cachectic patients *vs.* non-cachectic patients, $t=2{\cdot}13$ ($P<0{\cdot}05$) and for cachectic *vs.* tumour-free controls, $t=5{\cdot}42$ ($P<0{\cdot}001$). On the other hand, there seems to be no clear difference in mean total protein or fasting glucose levels between the 3 categories. Only 1/10 cachectic and 2/10 non-cachectic patients had raised levels of liver enzymes.

Table V compares the insulin response to glucagon challenge in cachectic and non-cachectic cancer patients. It can be seen that whereas glucagon consistently augmented the fasting insulin levels of non-cachectic cancer patients, it had only a marginal effect in cachectic tumour patients.

### DISCUSSION

The results demonstrate a definite association between impaired glucose tolerance and cachexia in neoplasia. Cachexia is, in turn, seen to be associated with hypoalbuminemia, a feature which has been observed by several previous workers (Ariel, 1949; Winzler, 1953; Peden *et al.*, 1957).

Alteration in the functional capacity of the liver (the major site of albumin synthesis) (Madden and Whipple, 1940) does not seem to be responsible for the low levels of circulating albumin observed in neoplastic cachexia (Winzler, 1953). Our data are consistent with this thesis. Hence, in the absence of any gastro-intestinal disturbance, chronic haemorrhage, or sepsis, hypoalbuminemia, in advanced cancer patients, is likely to arise from either increased protein breakdown, excessive utilization of protein by the tumour, reduced protein intake, or a combination of these. Several previous investigations have failed to demonstrate a protein-losing catabolic state in malignant cachexia (Waterhouse *et al.*, 1951; Fenninger and Mider, 1954; Holroyde *et al.*, 1975).

On the other hand, protein loss (in terms of nitrogen) experienced during tumour growth seems to follow a pattern very similar to that observed during dietary starvation. For example, the tissues and organs which lose nitrogen during starvation are the ones that lose nitrogen during tumour growth (Sherman *et al.*, 1950). Evidently, protein deprivation in advanced cancer seems to arise not only from anorexia, the major cause of cachexia (Theologides, 1972) but also from monopolization of the host's protein metabolic pool by the tumour tissue (Mider *et al.*, 1948). It follows that cancer patients suffer from protein malnutrition derived from both a low dietary intake and preferential utilization of protein metabolites by the tumour. The resulting protein-deficient state is hence analogous to that experimentally induced in normal weanling rats by Weinkove *et al.* (1976), since not only were these rats on a low protein diet, but they were in a phase of rapid growth, a state which in itself, utilizes a high level of protein.

The reason for impaired carbohydrate metabolism in the rat model appears to be a defect in the release of insulin from the pancreas. For example, insulin-releasing agents such as glucose and tolbutamide failed to elevate basal plasma insulin in the face of demonstrably adequate stores of pancreatic insulin (Weinkove *et al.*, 1976). This fact, taken in conjunction with our data on glucagon-mediated insulin release in cachectic cancer patients, suggests a similar underlying mechanism for glucose intolerance in human neoplasia.

From the foregoing discussion and the data presented above, the glucose intolerance exhibited by both cachectic cancer patients and protein-deficient weanling rats, seems to originate from a state of malnutrition due to reduced dietary intake of protein and increased protein demand. Hence, in order to improve the nutritional status of advanced cancer patients it may be necessary not only to increase their dietary intake but at the same time to somehow reduce the metabolic activity of tumour tissue. Failure to achieve these goals simultaneously may explain why, in the past, protein repletion alone has been unsuccessful in treating malignant cachexia, whilst similar treatment of non-malignant cachexia has resulted in a gain in weight and in total circulating albumin (Peden *et al.*, 1957).

### REFERENCES

Albano, J. D., Ekins, R. P., Maritz, G. & Turner, R. C. (1972) A sensitive, precise radioimmunoassay of serum insulin relying on charcoal separation of bound and free hormone moieties. *Acta Endocrinol.*, **70**, 487.

Ariel, I. M. (1949) The nature of postoperative hypoproteinemia in patients with gastro-intestinal cancer. *Surg. Gynecol. Obstet.*, **88**, 185.

Edwards, S. (1919) Blood sugar tolerance in cancer. *J. Indiana State Med. Assoc.*, **12**, 296.

Fenninger, L. D. & Mider, G. B. (1954) Energy and nitrogen metabolism in cancer. *Adv. Cancer Res.*, **2**, 229.

Glicksman, A. S. & Rawson, R. W. (1956) Diabetes and altered carbohydrate metabolism in patients with cancer. *Cancer*, **9**, 1127.

Grodsky, G. M., Bennett, L. L., Smith, D. F. & Schmid, F. G. (1967) Effect of pulse administration of glucose or glucagon on insulin secretion *in vivo*. *Metabolism*, **16**, 222.

Heard, C. R. C. (1966) Effects of severe protein-calorie deficiency on the endocrine control of carbohydrate metabolism. *Diabetes*, **15**, 78.

Holroyde, C. P., Gabuzda, T. G., Putnam, R. C., Pavie, P. & Reichard, G. A. (1975) Altered glucose metabolism in metastatic carcinoma. *Cancer Res.*, **35**, 3710.

Madden, S. C. & Whipple, G. H. (1940) Plasma proteins: their source, production and utilization. *Physiol. Rev.*, **20**, 194.

Marks, P. A. & Bishop, J. S. (1956) Alterations in carbohydrate metabolism associated with neoplasia in man. *Proc. Am. Ass. Cancer Res.*, **2**, 131.

Marks, P. A. & Bishop, J. S. (1957) Glucose metabolism in subjects with neoplastic disease: response to insulin and glucose tolerance. Follow-up studies. *Proc. Am. Ass. Cancer Res.*, **2**, 228.

Mider, G. B., Tesluk, H. & Morton, J. J. (1948) Effect of Walker carcinoma 256 on food intake, body-weight, and nitrogen metabolism on growing rats. *Acta Un. Int. Cancer*, **6**, 409.

Milner, R. D. G. (1971) Metabolic and hormonal responses to glucose and glucagon in patients with infantile malnutrition. *Pediatr. Res.*, **5**, 33.

Peden, J. C., Bond, L. F. & Maxwell, M. (1957) Comparative protein repletion in cancer and non-cancer cachexia. *Am. J. Clin. Nutr.*, **5**, 305.

Sherman, C. D. Jr., Morton, J. J. & Mider, G. B. (1950) Potential sources of tumour nitrogen. *Cancer Res.*, **10**, 374.

Smith, S. R., Edgar. P. J., Pozefsky, T., Chhetri, M. K. & Prout, T. E. (1975) Insulin secretion and glucose tolerance in adults with protein-calorie malnutrition. *Metabolism*, **24**, 1073.

Theologides, A. (1972) Pathogenesis of cachexia in cancer. A review and a hypothesis. *Cancer*, **29**, 484.

WATERHOUSE, C., FENNINGER, L. D. & KEUTMANN, E. H. (1951) Nitrogen exchange and calorie expenditure in patients with malignant neoplasms. *Cancer*, **4**, 500.

WEINKOVE, C., WEINKOVE, E. A. & PIMSTONE, B. L. (1976) Glucose tolerance and insulin release in malnourished rats. *Clin. Sci. Mol. Med.*, **50**, 153.

WINZLER, R. J. (1953) Plasma proteins in cancer. *Adv. Cancer Res.*, **1**, 503.


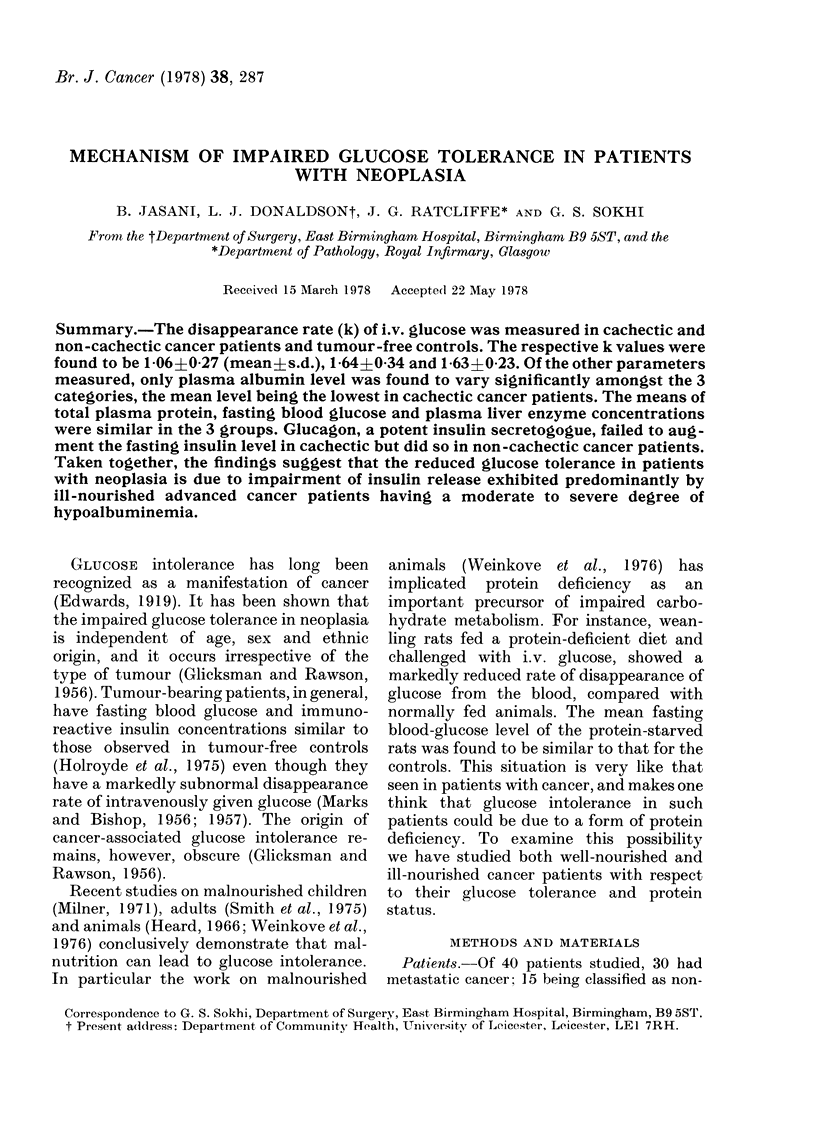

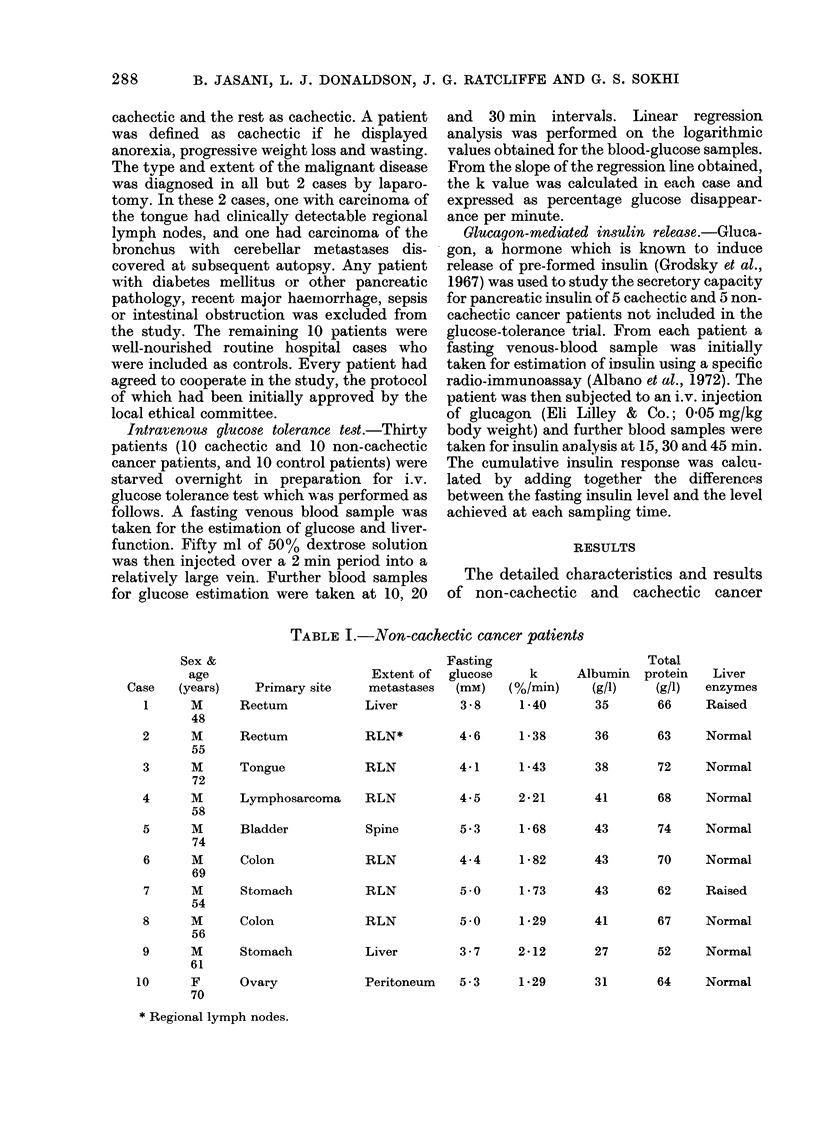

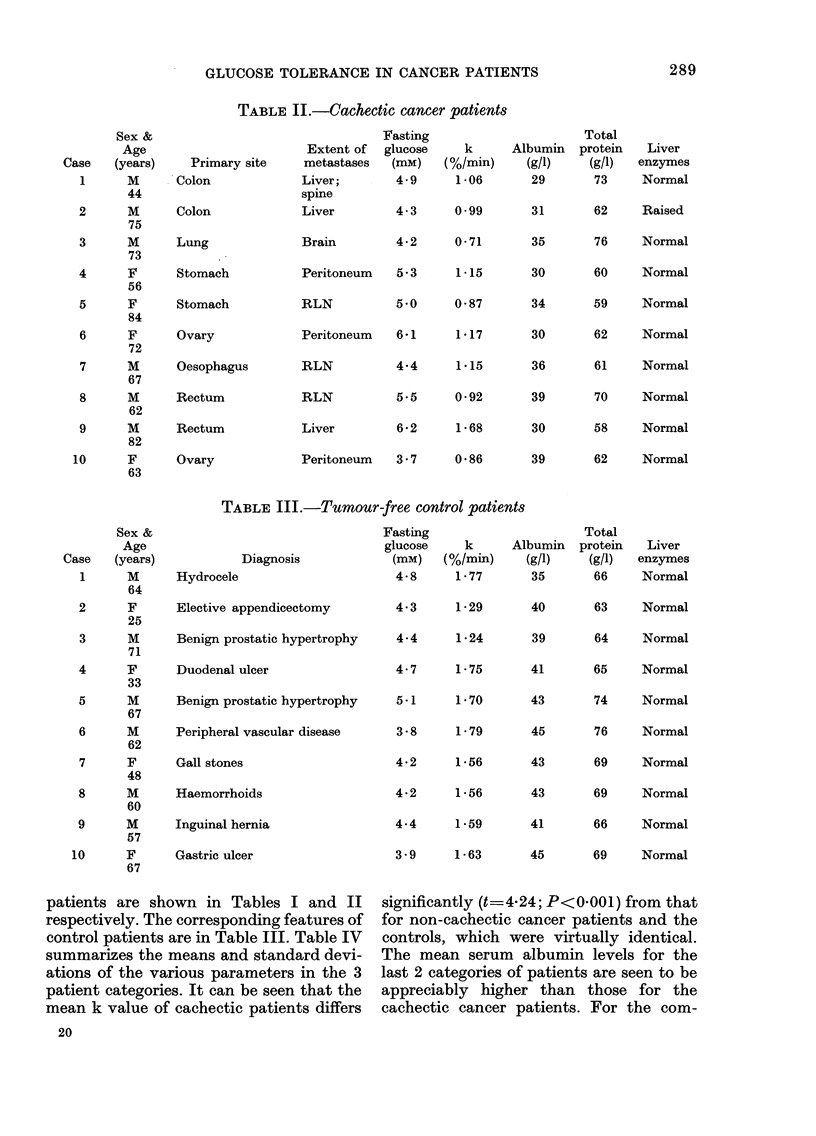

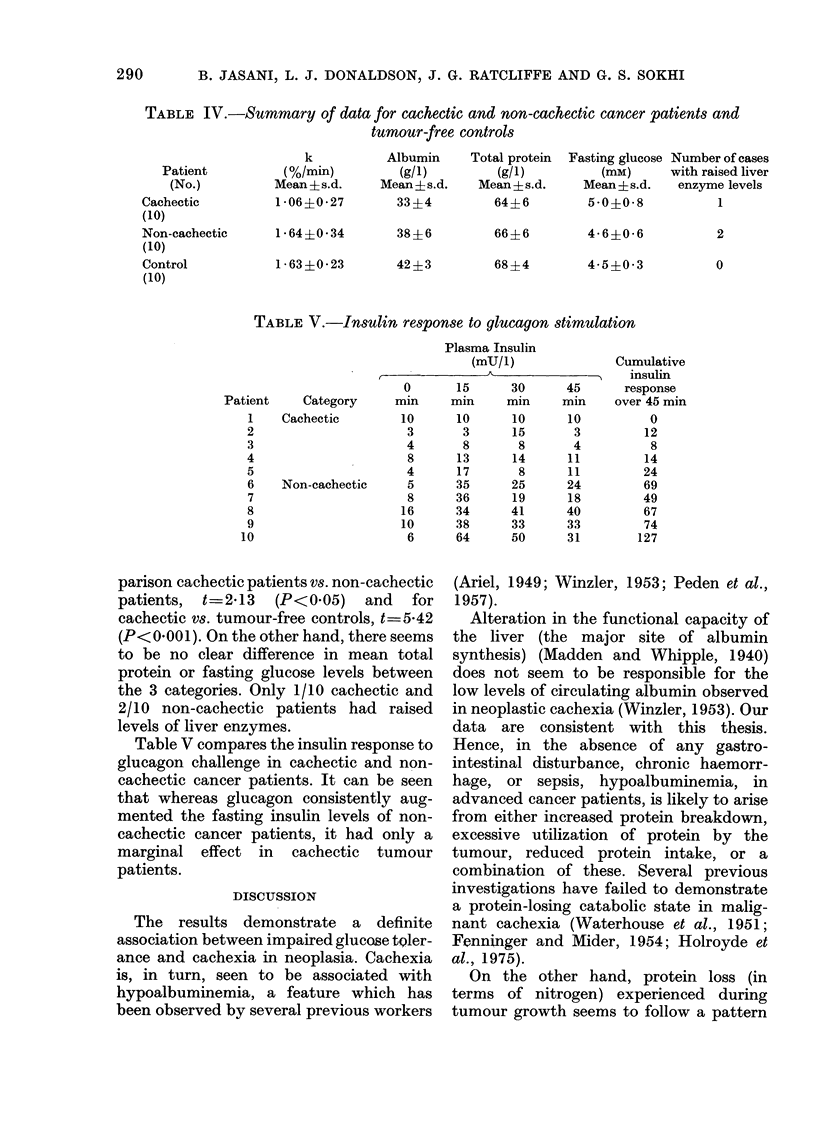

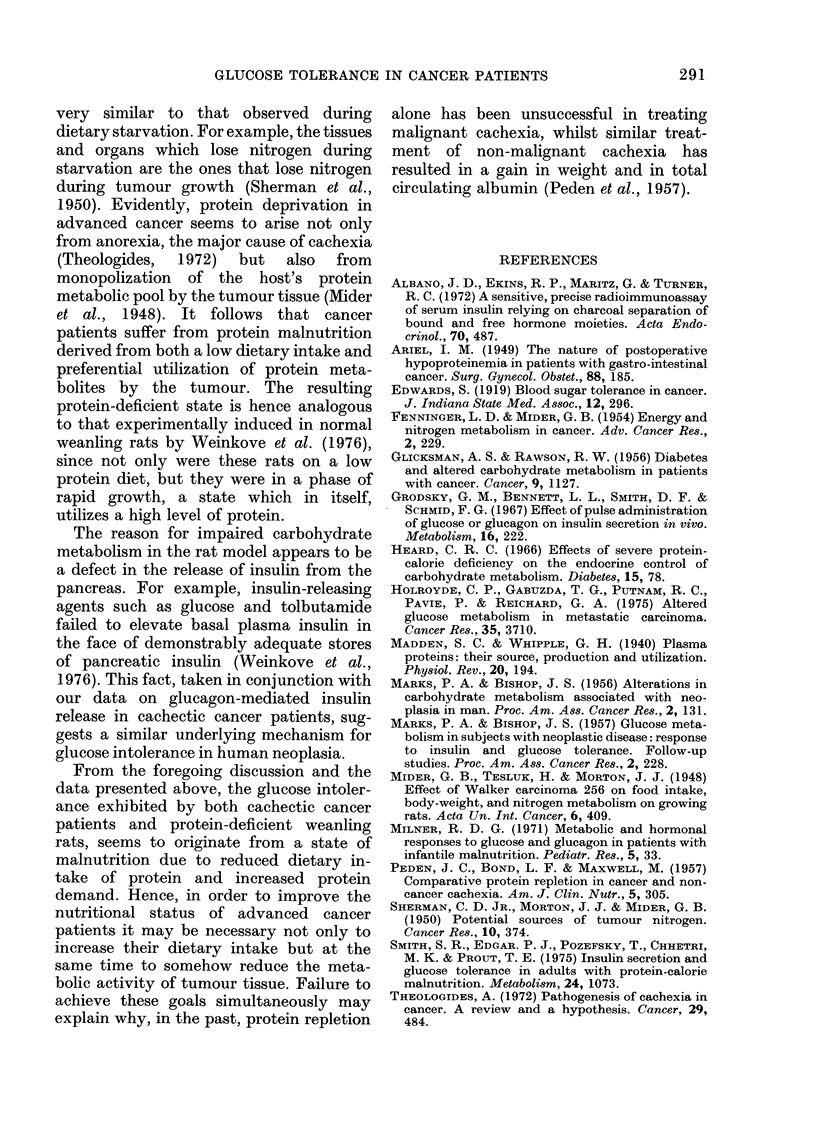

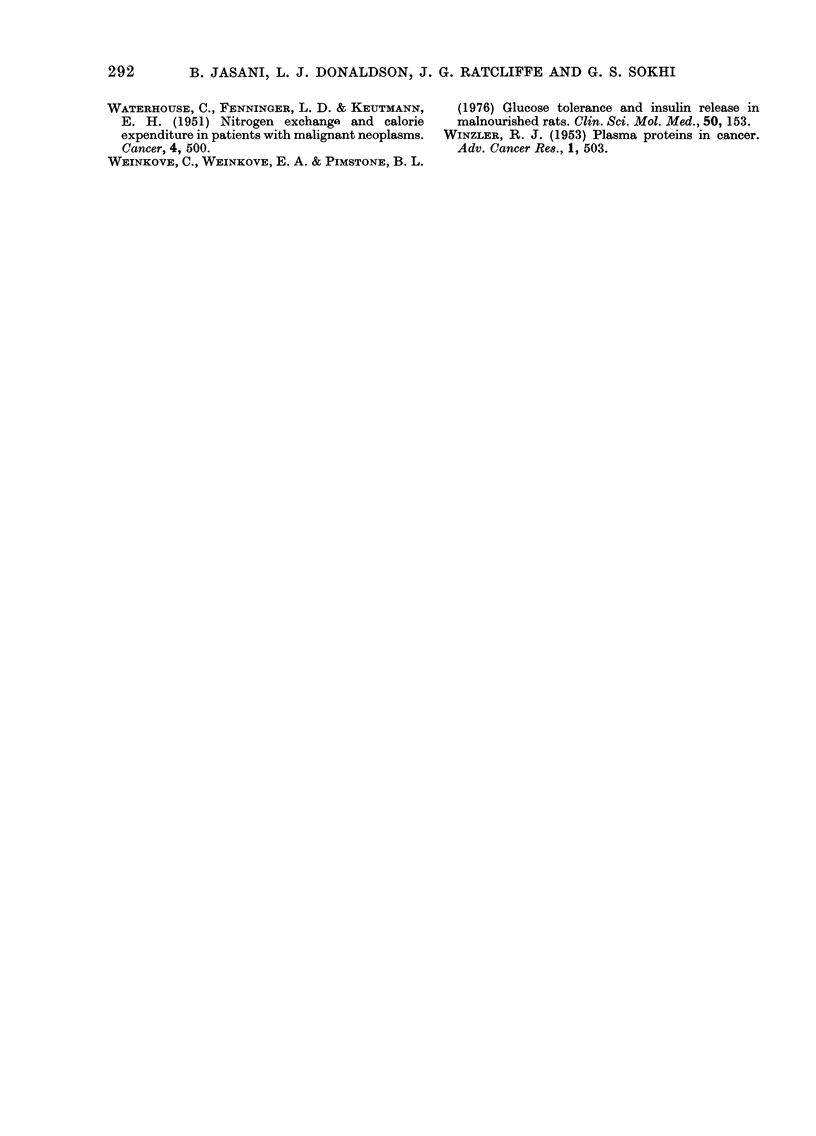


## References

[OCR_00838] Albano J. D., Ekins R. P., Maritz G., Turner R. C. (1972). A sensitive, precise radioimmunoassay of serum insulin relying on charcoal separation of bound and free hormone moieties.. Acta Endocrinol (Copenh).

[OCR_00854] FENNINGER L. D., MIDER G. B. (1954). Energy and nitrogen metabolism in cancer.. Adv Cancer Res.

[OCR_00859] GLICKSMAN A. S., RAWSON R. W. (1956). Diabetes and altered carbohydrate metabolism in patients with cancer.. Cancer.

[OCR_00864] Grodsky G. M., Bennett L. L., Smith D. F., Schmid F. G. (1967). Effect of pulse administration of glucose or glucagon on insulin secretion in vitro.. Metabolism.

[OCR_00870] Heard C. R. (1966). Effects of severe protein-calorie deficiency on the endocrine control of carbohydrate metabolism.. Diabetes.

[OCR_00875] Holroyde C. P., Gabuzda T. G., Putnam R. C., Paul P., Reichard G. A. (1975). Altered glucose metabolism in metastatic carcinoma.. Cancer Res.

[OCR_00907] PEDEN J. C., BOND L. F., MAXWELL M. (1957). Comparative protein repletion in cancer and non-cancer cachexia with special reference to changes in blood volume and total circulating plasma protein and hemoglobin.. Am J Clin Nutr.

[OCR_00912] SHERMAN C. D., MORTON J. J., MIDER G. B. (1950). Potential sources of tumor nitrogen.. Cancer Res.

[OCR_00917] Smith S. R., Edgar P. J., Pozefsky T., Chhetri M. K., Prout T. E. (1975). Insulin secretion and glucose tolerance in adults with protein-calorie malnutrition.. Metabolism.

[OCR_00923] Theologides A. (1972). Pathogenesis of cachexia in cancer. A review and a hypothesis.. Cancer.

[OCR_00930] WATERHOUSE C., FENNINGER L. D., KEUTMANN E. H. (1951). Nitrogen exchange and caloric expenditure in patients with malignant neoplasms.. Cancer.

[OCR_00941] WINZLER R. J. (1953). Plasma proteins in cancer.. Adv Cancer Res.

[OCR_00936] Weinkove C., Weinkove E. A., Pimstone B. L. (1976). Glucose tolerance and insulin release in malnourished rats.. Clin Sci Mol Med.

